# Recognition of LPS by TLR4: Potential for Anti-Inflammatory Therapies

**DOI:** 10.3390/md12074260

**Published:** 2014-07-23

**Authors:** Reindert Nijland, Tom Hofland, Jos A. G. van Strijp

**Affiliations:** Medical Microbiology, University Medical Center Utrecht, Heidelberglaan 100, 3584 CX Utrecht, The Netherlands; E-Mails: r.nijland@umcutrecht.nl (R.N.); t.hofland@students.uu.nl (T.H.)

**Keywords:** LPS, lipid A, TLR4, sepsis therapy

## Abstract

LPS molecules of marine bacteria show structures distinct from terrestrial bacteria, due to the different environment that marine bacteria live in. Because of these different structures, lipid A molecules from marine bacteria are most often poor stimulators of the Toll-like receptor 4 (TLR4) pathway. Due to their low stimulatory potential, these lipid A molecules are suggested to be applicable as antagonists of TLR4 signaling in sepsis patients, where this immune response is amplified and unregulated. Antagonizing lipid A molecules might be used for future therapies against sepsis, therapies that currently do not exist. In this review, we will discuss these differences in lipid A structures and their recognition by the immune system. The modifications present in marine lipid A structures are described, and their potential as LPS antagonists will be discussed. Finally, since clinical trials built on antagonizing lipid A molecules have proven unsuccessful, we propose to also focus on different aspects of the TLR4 signaling pathway when searching for new potential drugs. Furthermore, we put forward the notion that bacteria probably already produce inhibitors of TLR4 signaling, making these bacterial products interesting molecules to investigate for future sepsis therapies.

## 1. LPS

**Figure 1:**
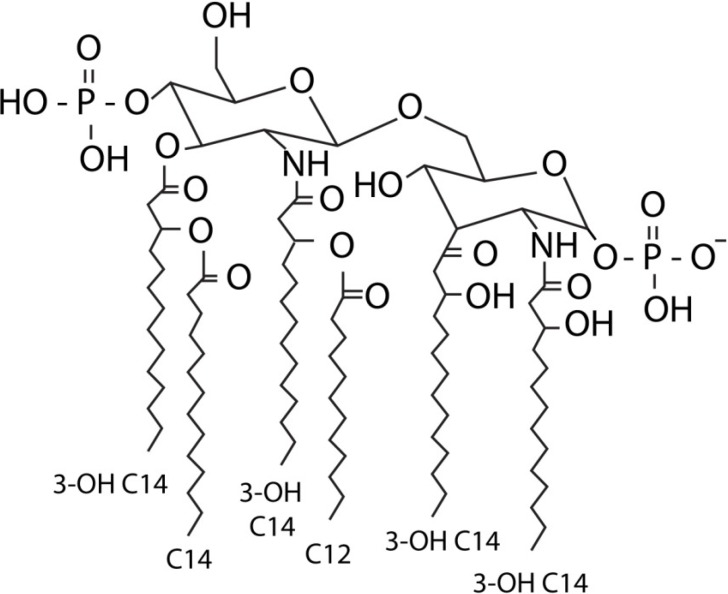
Structure of the *E. coli* lipid A molecule, which is regarded as the most potent immune stimulator.

The major component of the outer leaflet of the outer membrane of Gram-negative bacteria is lipopolysaccharide (LPS). These molecules are in direct contact with the outside environment and, as such, are thought to play a role in resistance against outside dangers [1]. LPS is essential for viability in almost all Gram-negative bacteria. One cell of the model organism for Gram-negative bacteria, *E. coli*, contains approximately 3.5 × 10^6^ LPS molecules [2]. LPS consists of three different parts, in the following order, from inside the membrane to the outside: lipid A, the core (sometimes subdivided into the inner and the outer core) and the O-antigen [2]. The normally hidden lipid A part (Figure 1) is highly immunogenic and is recognized to be responsible for the development of septic shock or sepsis, an amplified and unregulated immune response by the host, which eventually could lead to organ failure, coagulation abnormalities and death [3,4]. Currently, there are no specific drugs for LPS-induced clinical syndromes [5,6].

## 2. Immune Recognition of LPS through the TLR4 Pathway

**Figure 2:**
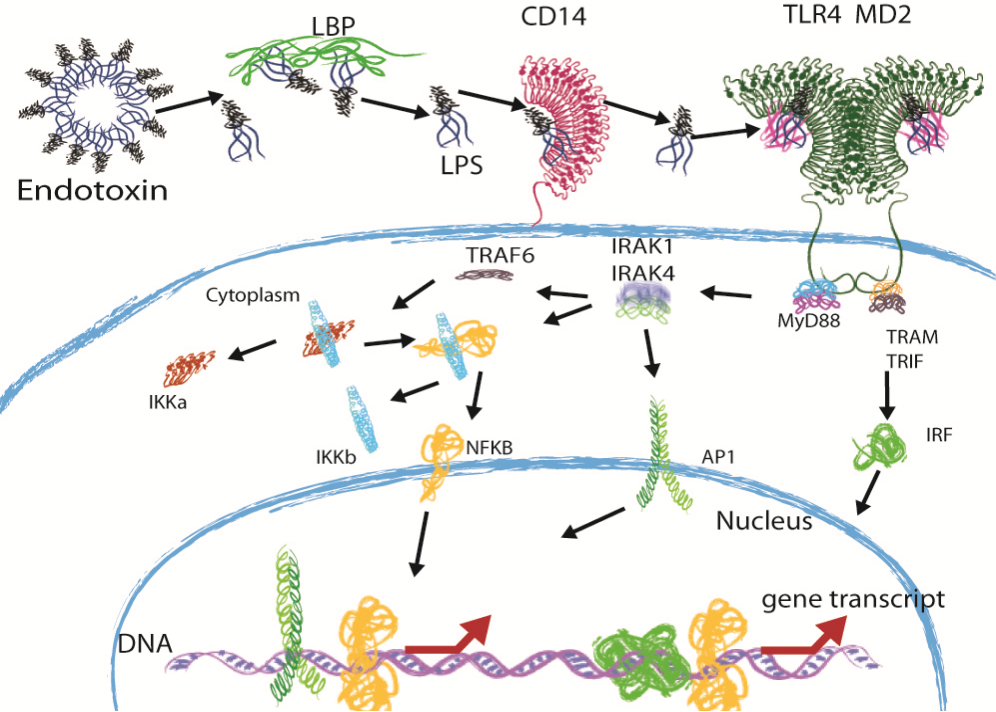
The TLR4 signaling pathway. When LPS is bound to the bacterial outer membrane or present in intact outer membrane vesicles, its toxic potential is not released, and as such, these molecules were termed “endotoxins”. As soon as LPS is released, this is changed. Free LPS is bound by LBP and transferred to CD14. MD-2 then binds the LPS and forms LPS-MD-2-TLR4 complexes. Dimerization of two of these complexes then occurs. Dimerization of TLR4 molecules leads to the recruitment of adapter molecules: MyD88, the TIR-domain containing adapter protein-inducing IFN-β (TRIF) and the TRIF-related adapter molecule (TRAM). A signaling cascade is initiated, which eventually leads to the degradation of the IKK complex, which frees the transcription factor, NF-κB. NF-κB then moves into the nucleus and starts transcription of pro-inflammatory cytokines, such as IL-6 and TNF-α. Via a different pathway, initiated by different adapter molecules, type I interferon genes are also transcribed, leading to the production of IFN-α/β.

The Lipid A part of LPS is not recognized by the host when it is anchored inside the bacterial outer membrane. When LPS is released, the lipid A part becomes exposed and initiates an immune response. The release of LPS from the membrane is caused by growth or cell lysis [4] A schematic overview of the immune recognition of LPS is given in Figure 2. The recognition of Lipid A starts with binding to lipopolysaccharide-binding protein (LBP), an acute phase protein. LBP then catalyzes the transfer of LPS to CD14 [4,6]. CD14 is a glycosyl-phosphatidylinositol (GPI)-linked receptor on monocytes, macrophages and polymorphonuclear leukocytes and binds LPS-LBP complexes. Because CD14 lacks transmembrane and cytoplasmic domains, it is thought not to have signaling capabilities [4,6]. These signaling capabilities are provided by Toll-like receptor 4 (TLR4) [7], in complex with myeloid-differentiation protein 2 (MD-2), which interacts with CD14. Both TLR4 and MD-2 are found to be essential for signaling [8,9,10]. Where rough (*R*-form) LPS requires LBP and CD14, smooth (*S*-form) LPS has been described to directly interact with TLR4 [11]. Upon binding of LPS to the TLR4-MD-2 complex, dimerization of this complex occurs, and signaling is initiated by the interaction of the intracellular TLR4 domains [6,10]. Dimerization leads to the recruitment of adapter molecules and, via a signaling cascade, eventually, to the activation of the transcription factor nuclear factor-κB (NF-κB), which leads to the production of pro-inflammatory cytokines, such as IL-6 and tumor necrosis factor-α (TNF-α) [6,12,13].

## 3. Sepsis and the Potential of Structurally Different LPS Molecules as Antagonists

Sepsis is a clinical syndrome that originates from the TLR4 response, and therefore, future therapies focus on the elements of this signaling pathway. The observation that not all LPS molecules produced by different bacteria induce the same immune response, and some not at all, has triggered research into the capability of certain LPS molecules to act as antagonists to treat sepsis in future therapies [5]. Because these LPS molecules are still able to bind MD-2 and TLR4, but not initiate the signaling pathway, they are able to compete for MD-2 and TLR4 binding sites with more potent inflammatory LPS molecules. Successful competition leads to an inhibition of TLR4 signaling and, therefore, a reduction of sepsis syndromes. Bacteria can produce many different forms of lipid A with their modification systems, which are mostly induced or repressed by the changes in growth conditions, such as pH, the presence of antimicrobial peptides and divalent cation concentrations [14]. It is shown that marine bacteria, which live in very different and harsh environments, with low temperature, high salt concentration and high hydrostatic pressure, produce different lipid A molecules. These have the potential to be used as antagonists in therapies against sepsis, when they show reduced immune stimulatory abilities [5,15].

## 4. Structure and Immune Recognition of *E. coli* Lipid A

In order to determine the consequences of structural differences in the lipid A molecule regarding immune recognition, a basic understanding of the TLR4-MD-2-LPS complex is required. The crystal structure of this complex was determined using an *E. coli* LPS [16], which is regarded as one of the most potent LPS molecules [17]. The *E. coli* lipid A molecule consists of a β-1,6-linked d-glucosamine disaccharide, which is acylated with six fatty acids and carries two phosphate molecules (see Figure 1) [17]. Five of these six fatty acids interact with a hydrophobic pocket of MD-2, while one fatty acid is partially exposed on the surface for hydrophobic interactions required for dimerization. The ester and amide groups that connect the fatty acids to the glucosamine backbone are also exposed to the surface of MD-2, and they interact with hydrophilic side chains on the MD-2 pocket, TLR4 and the second TLR4 molecule. The phosphate groups interact with positively-charged residues from MD-2 and both TLR4 molecules. In order to establish dimerization, binding of lipid A induces a structural shift of 5 A° in MD-2, which moves critical residues for interaction with the second TLR4 molecule into the right conformation [16]. Not only do all components of the lipid A interact with the MD-2-TLR4 complex, but many residues also interact with the second TLR4 molecule, thereby promoting dimerization [16]. The structure and interaction with the TLR4-MD-2 complex of the *E. coli* lipid A molecule will serve as the reference for other lipid molecules described below, and the effects on immune recognition by structural differences will be evaluated by comparing it to this lipid A.

## 5. Immune Recognition of Lipid A Structures of Other Terrestrial Bacteria

The effects of structural differences in lipid A structure on immune recognition are described below. The LPS molecule of *Acinetobacter baumannii* was found to be a very potent stimulator of TLR4 signaling, comparable to *E. coli* LPS [18]. The structure of the lipid A molecule was found to resemble the structure of *E. coli* LPS, except for one extra fatty acid chain [19,20]. This higher degree of acylation does not seem to influence immune recognition by the TLR4-MD-2 complex, showing that in the case of *A. baumannii*, an extra fatty acid does not abrogate LPS binding to the complex, and the LPS is still able to initiate signaling.

Although the most potent lipid A molecules contain six fatty acids, not all lipid A molecules with six fatty acids are recognized by TLR4. The lipid A molecules of *Leptospira interrogans* and *Legionella pneumophila* contain six fatty acids, but show other structural differences with the *E. coli* lipid A. The *L. interrogans* lipid A contains only one methylated phosphate group [21], and the *L. pneumophila* lipid A contains one large acyl chain of 27 of 28 carbon atoms [22]. It was described that LPS of *Leptospira interrogans* and *Legionella pneumophila* are not recognized by TLR4, but by TLR2 [23]. However, all observations in the literature describing the recognition of lipid A by TLR2 are now thought to be caused by contamination of the lipid A with lipoproteins, the direct activator of TLR2 [24,25,26]. Immune recognition of *Leptospira interrogans* lipid A by TLR4 is probably disturbed by the absence of negative-charged residues at the site of the phosphate groups, since these negative charges are important for TLR4 signaling [16]. Interestingly, the LPS of *Bordetella pertussis* (which signals via TLR4) was able to antagonize the effect of the *Legionella* lipid A molecules [22].

## 6. Decreased Lipid A Acylation Reduces Immune Potency

Although an extra fatty acid does not seem to influence immune recognition by TLR4 for the LPS of *Acinetobacter baumannii*, the decrease of acylation has long been recognized for its reduction in the immune potency of the lipid A molecule. An interesting example is the lipid A molecule of *Yersinia pestis*, which shifts its structure at different temperatures [27]. It was shown that the lipid A structure when bacteria were cultured at 27 °C was a mixture of many forms, ranging from tri-acyl to hexa-acyl, while the lipid A structure of bacteria cultured at 37 °C lacks hexa-acylated lipid A. The immune potency of the lipid A was reduced 100-times by the decrease of acylation at 37 °C [27]. The structural switch of lipid A at different temperatures is thought to be a mechanism of immune evasion, where a change of host (and thereby temperature) would result in the production of lipid A molecules that are poorly recognized [27]. The result of deacylation was also shown for other bacterial pathogens, like *Pseudomonas aeruginosa*, *Francisella tularensis*, *Bacteroides fragilis* and *Chlamydia trachomatis* [28]. De-acylated lipid A (either penta- or tetra-acylated lipid A) was shown to need 100-times the amount of LPS to induce the same response as the hexa-acylated structure [28].

The reduced potency of lipid A molecules with less than six acyl chains to stimulate TLR4-MD-2 complexes can be structurally explained. The fatty acid chains of penta- and tetra-acylated lipid A molecules are proposed to move further into the hydrophobic pocket of MD-2 to maximize hydrophobic contact. This should lead to substantially high energetic penalties when these fatty acids move back to the surface of MD-2 to interact with the second TLR4 molecule for dimerization [16]. Therefore, the dimerization reaction required for TLR4 signaling is energetically inhibited by lipid A molecules with five or less fatty acid chains. However, this mechanism is not true for all lipid A molecules with five fatty acids, like the lipid A from *Bordetella pertussis*, which is known to be a potent stimulator of TLR4 signaling [22].

Not only the amount of fatty acids, but also the length of the fatty acids has been investigated [29]. In this study, synthetic lipid A mimetic compounds were created, which varied in their length of the secondary acyl chains. All compounds were hexa-acylated to mimic the most potent structure of lipid A. It was found that fatty acid chains with a length of eight carbon atoms were required for TLR4 stimulation, and a length of 10 carbon atoms was optimal [29]. The switch of a fatty acid chain with a length of 10 carbon atoms to a chain of six carbon atoms diminished the potency of the synthetic lipid A mimetic compounds [29]. Interestingly, it was suggested that CD14 is able to enhance the responsiveness to lipid A molecules with a suboptimal length (eight, 12 and 14 carbon atoms) to create immune recognition of a greater variety of lipid A molecules [29].

## 7. Immune Recognition of Lipid A Structures from Marine Bacteria

In order to evaluate marine lipid A structures for their ability to antagonize LPS signaling, the focus on their structure should be on both the number and length of their fatty acid chains and the presence of phosphate groups, since these features of the lipid A molecule seem to be most important for TLR4 recognition. If marine lipid A molecules show low immune stimulatory effects, but are still able to bind MD-2 and TLR4 binding sites, they could be used to compete with normal LPS molecules that are the cause of sepsis. An overview of the marine lipid A structures is given in Table 1.

**Table 1:** Overview of the lipid A structures from marine bacteria.

Species	No. of Acyl Chains	No. of Phosphorylations	Reference
*Pseudoalteromonas haloplanktis* (TAC 125)	5	2	[30]
*Pseudoalteromonas haloplanktis* (ATCC 14393)	5	2	[1]
*Alteromonas addita*	5	2	[31]
*Marinomonas vaga*	5	1	[32]
*Pseudoalteromonas issachenkonii*	4 (5)	2	[33]
*Alteromonas macleodii*	4 (5)	2	[34]
*Synechococcus* strains CC9311 and WH8102	4	0	[35]
*Shewanella pacifica*	6	2	[36]
*Chryseobacterium scophtalmum*	2	1	[1]
*Marinomonas communis*	5	1	[15]
*Marinomonas mediterranea*	5 (6)	2	[15]

Most of the marine Gram-negative bacteria show lipid A molecules that have lower levels of acylation, like penta-acylated (*Pseudoalteromonas*, *Alteromonas* and *Marinomonas* strains) or tetra-acylated lipid A molecules (*Pseudoalteromonas* and *Alteromonas* strains). Furthermore, next to a lower degree of acylation, most marine bacteria contain fatty acid chains that are also relatively short compared to terrestrial bacteria. Finally, the degree of phosphorylation is also lower in some marine lipid A structures, with some species containing only one phosphorylation and others even none at all (e.g., *Marinomonas* and *Synechococcus* strains). The most unusual lipid A molecule discovered is that of *Chryseobacterium scophtalmum*. This lipid A molecule was found to be a monosaccharide, mono-phosphorylated, with two fatty acid chains of 15 and 17 carbon atoms long [1]. This molecule showed resemblance to the precursor of *E coli* lipid A, which is called lipid X [1].

Some of these marine lipid A molecules have been tested for their ability to stimulate TLR4 signaling. For example, the toxicity of the *Marinomonas vaga* lipid A molecule was tested in sensitized mice, and it was found that the lethal dose of this lipid A molecule was more than 20-times higher than the lipid A from *Yersinia pseudotuberculosis*, thus showing a lower immune potency [32]. The marine lipid A molecules from *Marinomonas communis*, *Marinomonas mediterranea* and *Chryseobacterium scophtalmum* were also tested for their ability to stimulate TLR4 signaling [15]. All LPS molecules showed a lower toxicity in sensitized mice compared to *E coli* and *Yersinia pseudotuberculosis* (hexa-acylated) LPS [15]. All of the LPS molecules studied were weak stimulators of immune cells, leading to less release of inflammatory cytokines, like TNF-α [15]. One of the lipid A molecules, from *Marinomonas communis*, was also shown to inhibit the cytokine induction by *E. coli* LPS, showing its antagonistic potential by competing for LPS binding sites [15]. More recently, lipid A molecules from several *Pseudoalteromonas* strains were tested for their antagonistic properties [37]. These lipid A molecules were also shown to have low immune stimulatory potential, as 100-times more was needed to stimulate immune cells, and still, the same concentrations of IL-6 and TNF-α as *E. coli* LPS could not be induced [37]. Furthermore, lipid A molecules from these marine bacteria were able to dose-dependently inhibit the stimulation of immune cells by *E. coli* by blocking TLR4 activation, showing their antagonistic potential, as well [37]. Interestingly, this inhibition was not visible when looking at TNF-α induction, leaving the authors to speculate that *E. coli* can induce the expression of TNF-α via pathways other than TLR4 [37].

## 8. Main Differences between Marine and Terrestrial Lipid A Structures

Together, these data show that lipid A molecules from marine bacteria show distinct structures from terrestrial bacteria. Overall, lipid A molecules from marine bacteria show a lower degree of acylation and phosphorylation, which are properties known to affect TLR4 recognition [16]. Due to these chemical differences, the lipid A molecules show reduced immune stimulatory potential and toxicity [32] and even the ability to antagonize potent lipid A molecules *in vitro*, like those from *E. coli* [15,37]. Lipid A from marine bacteria has the ability to antagonize potent lipid A molecules from bacteria that are the causative agents of sepsis and septic shock, which make them potential targets to use for therapies against these syndromes, therapies that are, to date, still not present [5].

## 9. Why Do Marine Bacteria Have Different Lipid A?

Changes in temperature affect membrane fluidity; at lower temperatures, the membrane is less fluid [38]. It was shown for *E. coli* that it rapidly modifies its LPS when it is transferred from normal growing temperatures (>30 °C) to 12 °C [39]. To have optimal outer membrane fluidity, marine bacteria, especially of colder seas, are likely to have adapted their LPS to these conditions. Especially low acylation is a distinctive feature of bacteria growing in cold environments [40]. Not only temperature, but also pressure and salt concentrations found in the marine habitat differ from that of most terrestrial niches. Adaptations of the outer membrane will shield the cells from these natural stress factors [1].

The adaptations in the lipid A of marine bacteria are relevant for survival in the marine environment. Pathogenic bacteria infecting humans do not face the low temperature and high pressures of the ocean, but specifically adapt their membrane composition to evade the host immune system. Although lipid A from marine bacteria can be a potent antagonist of TLR4 signaling, this effect will be coincidental, as no selective pressure has shaped these interactions. It is more likely that lipid A molecules from Gram-negative human pathogens have evolved these properties. However, as we will discuss below, these pathogen-specific lipid A molecules are modified sufficiently to reduce or prevent *in vivo* immune activation, but are still too potent to be effective systemic anti-sepsis agents.

## 10. Drugs Based on Modified LPS in Sepsis Therapy Have Limited Success

The potential of TLR4 antagonists for sepsis therapy has long been recognized. Therefore, several potential drugs have entered clinical trials to test their efficacy against sepsis. One of these was a lipid A analogue, called Eritoran (E5564), which entered a Phase III clinical trial. The structure of Eritoran is based on the lipid A molecule of *Rhodobacter sphaeroides*, which is a non-pathogenic lipid A [41,42]. It was shown that Eritoran was able to block responses by human monocytes to LPS, even in nanomolar concentrations, making it a powerful antagonist with high potential to be used for therapy [43]. Later, it was shown that Eritoran binds the hydrophobic pocket of MD-2, without any interaction with TLR4. Therefore, Eritoran could compete with LPS for the binding site of MD-2 without leading to TLR4 dimerization, and consequently, Eritoran inhibited TLR4 activation [42]. Because of these promising features, Eritoran entered clinical trials. However, the Phase III clinical trial was halted, due to a lack of significant results for Eritoran administration in 2000 sepsis patients [44]. Although this was in contrast with the results of earlier trials, the trial showed that Eritoran treatment had no significant benefit for sepsis patients [44].

Another TLR4 antagonist that entered clinical trials is TAK-242. This chemical compound binds the intracellular domain of TLR4 and is, thus, not a real LPS antagonist [45]. However, TAK-242 was able to inhibit the production of TNF-α, IL-1β and IL-6 in mice, even if these cytokine levels were already high when TAK-242 was administered [45]. Again, just like Eritoran, the Phase III clinical trial of TAK-242 was halted, because the compound was unable to suppress cytokine levels in patients with severe sepsis [41].

## 11. Bacterial TLR4 Inhibitors as Therapeutics against Sepsis

In spite of the potential of these and other LPS and/or TLR4 antagonists *in vitro* and in Phase I and II clinical trials, none of them have been shown to be effective for sepsis therapy, and new strategies should be evaluated. In the search for new potential drug agents for sepsis therapy, we propose to search in a so-far under-explored place: the pathogenic bacteria itself. Bacteria are known to produce many immune evasion molecules that act on different parts of the innate immune system, like the complement system, antimicrobial peptides and TLRs [46,47]. For example, *Pseudomonas aeruginosa* was shown to produce a protease that cleaves monomeric flagellin, the ligand for TLR5 [48]. The destruction of the TLR5 ligand leads to the inhibition of TLR5 signaling and, therefore, results in immune evasion. The same protease was also able to cleave C2 and, therefore, block complement activation [49]. Another example comes from *Staphylococcus aureus*, which produces an immune evasion protein, called SSL3, which specifically inhibits TLR2, likely by competing for ligand binding [50]. The advantage of using proteins from bacteria is that these proteins are shaped through natural selection for a specific goal; the species that produces proteins with the highest inhibitory effect has a greater chance of surviving within its host. Furthermore, immune evasion by bacteria does not rely on one single point of action. For example, *Staphylococcus aureus* has been shown to inhibit the complement cascade at almost every step required for complement activation [46,47]. The same principle could be true for TLR4 signaling. Every signaling molecule active in the TLR4 signaling pathway (Figure 2) can be targeted by bacteria to abrogate the signal. In the search of new drug agents, we suggest to also look further down the pathway of TLR4 signaling than just the interaction with the receptor. Compounds that target other signaling proteins may be more potent inhibitors. Since Gram-positive bacteria do not contain LPS, they are unlikely to be the best candidates to look for antagonists of the TLR4 signaling. Therefore, to identify these antagonist, one should turn to the Gram-negative bacteria, preferably human pathogens.

## 12. Compounds that Target the TLR4 Signaling Pathway

Recently, several chemical compounds have been tested for their ability to inhibit TLR4 signaling without directly interacting with the TLR4 receptor. One of these is an antibody, called WN1 222-5. This antibody has been shown to inhibit the inflammatory response *in vivo* and *in vitro*, showing a potential use in future sepsis therapies [51]. It was shown that this antibody does not bind the lipid A moiety of LPS, but it binds to the inner core of the LPS molecule [51]. Interestingly, it was shown that the binding of LPS by WN1 222-5 mimics the binding of LPS by TLR4 [51]. Two compounds have been reported to interfere TLR4 signaling by targeting MD-2. One is an arylidene malonate derivative, which was able to block LPS-induced cytokine production [52]. The predicted binding site of this compound was inside the LPS binding site of the TLR4-MD-2 complex, making it able to disrupt protein-protein interactions between TLR4 and MD-2 (the actual structure of binding has not been determined) [52]. Another compound shown to be able to bind MD-2 is paclitaxel, an anticancer drug that induces cell cycle arrest and cell death in cancer [53]. Paclitaxel was shown to improve animal survival after admission of a lethal dose of LPS, to reduce cytokine levels in LPS-treated mice and to reduce NF-κB activation [53]. The mechanism of action was shown to be binding to MD-2, which reduced interaction with TLR4 [53]. A different strategy was used by another research group, which used TRAM-derived decoy peptides [54]. Two of these peptides were shown to inhibit TLR4 signaling by blocking the recruitment of adapter proteins to the cytoplasmic domain of TLR4 [54]. These peptides were also shown to be able to block TLR4 signaling *in vivo*, showing the potential to be used in future therapies against sepsis [54]. Arsenic trioxide was found to block TLR4 signaling via the TRIF-dependent pathway only, although a clear mechanism of action has not been determined [55].

## 13. Future Inhibitors of the TLR4 Signaling Pathway

The examples above show that TLR4 signaling can be inhibited in several different ways, using different kinds of molecules. Since clinical trials using lipid A analogues have not been successful, we propose that it is time to focus on different aspects of the TLR4 signaling pathway. This will bring more potential therapeutic targets for therapy and, with that, the ability to use more strategies to find drugs that are effective in sepsis therapy.

## 14. Concluding Remarks

Lipid A structures from marine bacteria show differences from terrestrial bacteria, most noteworthy a decrease in acylation and shorter fatty acid chains. These features make marine lipid A molecules less potent inducers of TLR4 signaling and even give them the potential to antagonize immune stimulatory lipid A molecules. However, the use of modified LPS antagonists in sepsis therapy has so far been unsuccessful. An explanation for this lack of *in vivo* efficacy may lie in the concentration needed for effective antagonistic effects at the infection site. There is a clear difference between evasion of recognition, as is used by many pathogens, and antagonistic activity against other more potent lipid A molecules. Modified lipid A with a 100-fold lower stimulatory capacity will still cause the same inflammation when administered at a 100-times higher concentration. For effective antagonism to be achieved at the site of infection, the dose that has to be administered systemically to the sepsis patient has to be much higher than 100× the concentration present at the infective site. A dose that is not lethal to the patient is unlikely to sufficiently inhibit the LPS present in the body of the patient.

Many marine bacteria contain LPS, which differs from the *E. coli* prototype, since the marine environment forces bacteria to have altered outer membranes. Therefore, exploring marine bacteria will be an obvious choice to screen for lipid A with properties attractive for developing anti-sepsis treatment. However, for these molecules to be successful as antagonists, their antagonistic potency needs to be at least one order of magnitude better compared to the best ones identified so far. Still, a good place to look for these would be in a cold and deep marine environment.

We also put forward that bacteria are likely to produce other products that inhibit TLR4 signaling. In the context of TLR4 signaling, these immune evasion molecules are likely to be found in Gram-negative pathogens. Additionally, since many Gram-negative pathogens are able to also directly inject proteins inside the cytosol of host cells using type III secretion systems [56], also immune evasion processes targeting the intracellular part of this pathway are not to be ignored. The successful identification of such proteins will provide a broader range of targets to inhibit uncontrolled immune activation during sepsis.

## References

[1] Leone S., Silipo A., Nazarenko E.L., Lanzetta R., Parrilli M., Molinaro A. (2007). Molecular structure of endotoxins from Gram-negative marine bacteria: An update. Mar. Drugs.

[2] Rietschel E.T., Kirikae T., Schade F.U., Mamat U., Schmidt G., Loppnow H., Ulmer A.J., Zähringer U., Seydel U., di Padova F. (1994). Bacterial endotoxin: Molecular relationships of structure to activity and function. FASEB J..

[3] Cohen J. (2002). The immunopathogenesis of sepsis. Nature.

[4] Van Amersfoort E.S., van Berkel T.J., Kuiper J. (2003). Receptors, mediators, and mechanisms involved in bacterial sepsis and septic shock. Clin. Microbiol. Rev..

[5] Solov’eva T., Davydova V., Krasikova I., Yermak I. (2013). Marine compounds with therapeutic potential in Gram-negative sepsis. Mar. Drugs.

[6] Fitzgerald K.A., Rowe D.C., Golenbock D.T. (2004). Endotoxin recognition and signal transduction by the TLR4/MD2-complex. Microbes Infect./Inst. Pasteur.

[7] Poltorak A., He X., Smirnova I., Liu M.-Y., Huffel C.V., Du X., Birdwell D., Alejos E., Silva M., Galanos C. (1998). Defective lps signaling in C3H/HeJ and C57BL/10ScCr mice: Mutations in TLR4 gene. Science.

[8] Gioannini T.L., Teghanemt A., Zhang D., Coussens N.P., Dockstader W., Ramaswamy S., Weiss J.P. (2004). Isolation of an endotoxin-MD-2 complex that produces Toll-like receptor 4-dependent cell activation at picomolar concentrations. Proc. Natl. Acad. Sci. USA.

[9] Da Silva Correia J., Soldau K., Christen U., Tobias P.S., Ulevitch R.J. (2001). Lipopolysaccharide is in close proximity to each of the proteins in its membrane receptor complex: Transfer from CD14 to TLR4 and MD-2. J. Biol. Chem..

[10] DeMarco M.L., Woods R.J. (2011). From agonist to antagonist: Structure and dynamics of innate immune glycoprotein MD-2 upon recognition of variably acylated bacterial endotoxins. Mol. Immunol..

[11] Huber M., Kalis C., Keck S., Jiang Z., Georgel P., Du X., Shamel L., Sovath S., Mudd S., Beutler B. (2006). R-form LPS, the master key to the activation ofTLR4/MD-2-positive cells. Eur. J. Immunol..

[12] Kagan J.C., Su T., Horng T., Chow A., Akira S., Medzhitov R. (2008). Tram couples endocytosis of Toll-like receptor 4 to the induction of interferon-β. Nat. Immunol..

[13] Tanimura N., Saitoh S., Matsumoto F., Akashi-Takamura S., Miyake K. (2008). Roles for LPS-dependent interaction and relocation of TLR4 and tram in TRIF-signaling. Biochem. Biophys. Res. Commun..

[14] Raetz C.R., Reynolds C.M., Trent M.S., Bishop R.E. (2007). Lipid a modification systems in Gram-negative bacteria. Annu. Rev. Biochem..

[15] Vorobeva E.V., Krasikova I.N., Solov’eva T.F. (2006). Influence of lipopolysaccharides and lipids a from some marine bacteria on spontaneous and *Escherichia coli* LPS-induced TNF-alpha release from peripheral human blood cells. Biochem. Biokhimiia.

[16] Park B.S., Song D.H., Kim H.M., Choi B.S., Lee H., Lee J.O. (2009). The structural basis of lipopolysaccharide recognition by the TLR4-MD-2 complex. Nature.

[17] Schromm A.B., Brandenburg K., Loppnow H., Moran A.P., Koch M.H., Rietschel E.T., Seydel U. (2000). Biological activities of lipopolysaccharides are determined by the shape of their lipid a portion. Eur. J. Biochem..

[18] Erridge C., Moncayo-Nieto O.L., Morgan R., Young M., Poxton I.R. (2007). *Acinetobacter baumannii* lipopolysaccharides are potent stimulators of human monocyte activation via Toll-like receptor 4 signalling. J. Med. Microbiol..

[19] Leone S., Sturiale L., Pessione E., Mazzoli R., Giunta C., Lanzetta R., Garozzo D., Molinaro A., Parrilli M. (2007). Detailed characterization of the lipid a fraction from the nonpathogen *Acinetobacter radioresistens* strain s13. J. Lipid Res..

[20] Pelletier M.R., Casella L.G., Jones J.W., Adams M.D., Zurawski D.V., Hazlett K.R., Doi Y., Ernst R.K. (2013). Unique structural modifications are present in the lipopolysaccharide from colistin-resistant strains of *Acinetobacter baumannii*. Antimicrob. Agents Chemother..

[21] Zahringer U., Lindner B., Inamura S., Heine H., Alexander C. (2008). Tlr2—Promiscuous or specific? A critical re-evaluation of a receptor expressing apparent broad specificity. Immunobiology.

[22] Girard R., Pedron T., Uematsu S., Balloy V., Chignard M., Akira S., Chaby R. (2003). Lipopolysaccharides from legionella and rhizobium stimulate mouse bone marrow granulocytes via Toll-like receptor 2. J. Cell Sci..

[23] Werts C., Tapping R.I., Mathison J.C., Chuang T.H., Kravchenko V., Saint Girons I., Haake D.A., Godowski P.J., Hayashi F., Ozinsky A. (2001). Leptospiral lipopolysaccharide activates cells through a TLR2-dependent mechanism. Nat. Immunol..

[24] Hirschfeld M., Ma Y., Weis J.H., Vogel S.N., Weis J.J. (2000). Cutting edge: Repurification of lipopolysaccharide eliminates signaling through both human and murine Toll-like receptor 2. J. Immunol..

[25] Hellman J., Tehan M.M., Shaw Warren H. (2003). Murein lipoprotein, peptidoglycan-associated lipoprotein, and outer membrane protein a are present in purified rough and smooth lipopolysaccharides. J. Infect. Dis..

[26] Lee H.-K., Lee J., Tobias P.S. (2002). Two lipoproteins extracted from *Escherichia coli* K-12 LCD25 lipopolysaccharide are the major components responsible for Toll-like receptor 2-mediated signaling. J. Immunol..

[27] Kawahara K., Tsukano H., Watanabe H., Lindner B., Matsuura M. (2002). Modification of the structure and activity of lipid a in *Yersinia pestis* lipopolysaccharide by growth temperature. Infect. Immun..

[28] Hajjar A.M., Ernst R.K., Tsai J.H., Wilson C.B., Miller S.I. (2002). Human Toll-like receptor 4 recognizes host-specific LPS modifications. Nat. Immunol..

[29] Stover A.G., da Silva Correia J., Evans J.T., Cluff C.W., Elliott M.W., Jeffery E.W., Johnson D.A., Lacy M.J., Baldridge J.R., Probst P. (2004). Structure-activity relationship of synthetic Toll-like receptor 4 agonists. J. Biol. Chem..

[30] Corsaro M.M., Piaz F.D., Lanzetta R., Parrilli M. (2002). Lipid a structure of *Pseudoalteromonas haloplanktis* TAC 125: Use of electrospray ionization tandem mass spectrometry for the determination of fatty acid distribution. J. Mass Spectrom..

[31] Leone S., Molinaro A., Sturiale L., Garozzo D., Nazarenko E.L., Gorshkova R.P., Ivanova E.P., Shevchenko L.S., Lanzetta R., Parrilli M. (2007). The outer membrane of the marine Gram-negative bacterium *Alteromonas addita* is composed of a very short-chain lipopolysaccharide with a high negative charge density. Eur. J. Org. Chem..

[32] Krasikova I.N., Kapustina N.V., Isakov V.V., Dmitrenok A.S., Dmitrenok P.S., Gorshkova N.M., Solov’eva T.F. (2004). Detailed structure of lipid a isolated from lipopolysaccharide from the marine *Proteobacterium marinomonas* vaga ATCC 27119. Eur. J. Biochem..

[33] Silipo A., Leone S., Lanzetta R., Parrilli M., Sturiale L., Garozzo D., Nazarenko E.L., Gorshkova R.P., Ivanova E.P., Gorshkova N.M. (2004). The complete structure of the lipooligosaccharide from the halophilic bacterium *Pseudoalteromonas issachenkonii* KMM 3549 T. Carbohydr. Res..

[34] Liparoti V., Molinaro A., Sturiale L., Garozzo D., Nazarenko E.L., Gorshkova R.P., Ivanova E.P., Shevcenko L.S., Lanzetta R., Parrilli M. (2006). Structural analysis of the deep rough lipopolysaccharide from gram negative bacterium alteromonas macleodii atcc 27126t: The first finding of?-kdo in the inner core of lipopolysaccharides. Eur. J. Org. Chem..

[35] Snyder D.S., Brahamsha B., Azadi P., Palenik B. (2009). Structure of compositionally simple lipopolysaccharide from marine Synechococcus. J. Bacteriol..

[36] Silipo A., Leone S., Molinaro A., Sturiale L., Garozzo D., Nazarenko E.L., Gorshkova R.P., Ivanova E.P., Lanzetta R., Parrilli M. (2005). Complete structural elucidation of a novel lipooligosaccharide from the outer membrane of the marine bacterium *Shewanella pacifica*. Eur. J. Org. Chem..

[37] Maaetoft-Udsen K., Vynne N., Heegaard P.M., Gram L., Frøkiær H. (2013). Pseudoalteromonas strains are potent immunomodulators owing to low-stimulatory LPS. Innate Immun..

[38] Sinensky M. (1974). Homeoviscous adaptation—A homeostatic process that regulates the viscosity of membrane lipids in *Escherichia coli*. Proc. Natl. Acad. Sci. USA.

[39] Carty S.M., Sreekumar K.R., Raetz C.R.H. (1999). Effect of cold shock on lipid a biosynthesis *Inescherichia coli*: Induction at 12 degrees C of an acyltransferase specific for palmitoleoyl-acyl carrier protein. J. Biol. Chem..

[40] Ramos J.L., Gallegos M.T., Marqués S., Ramos-González M.I., Espinosa-Urgel M., Segura A. (2001). Responses of Gram-negative bacteria to certain environmental stressors. Curr. Opin. Microbiol..

[41] Peri F., Piazza M. (2011). Therapeutic targeting of innate immunity with Toll-like receptor 4 (TLR4) antagonists. Biotechnol. Adv..

[42] Kim H.M., Park B.S., Kim J.I., Kim S.E., Lee J., Oh S.C., Enkhbayar P., Matsushima N., Lee H., Yoo O.J. (2007). Crystal structure of the TLR4-MD-2 complex with bound endotoxin antagonist eritoran. Cell.

[43] Czeslick E., Struppert A., Simm A., Sablotzki A. (2006). E5564 (eritoran) inhibits lipopolysaccharide-induced cytokine production in human blood monocytes. Inflamm. Res..

[44] Opal S.M., Laterre P.F., Francois B., LaRosa S.P., Angus D.C., Mira J.P., Wittebole X., Dugernier T., Perrotin D., Tidswell M. (2013). Effect of eritoran, an antagonist of MD2-TLR4, on mortality in patients with severe sepsis: The access randomized trial. JAMA.

[45] Takashima K., Matsunaga N., Yoshimatsu M., Hazeki K., Kaisho T., Uekata M., Hazeki O., Akira S., Iizawa Y., Ii M. (2009). Analysis of binding site for the novel small-molecule TLR4 signal transduction inhibitor TAK-242 and its therapeutic effect on mouse sepsis model. Br. J. Pharmacol..

[46] Rooijakkers S.H., van Strijp J.A. (2007). Bacterial complement evasion. Mol. Immunol..

[47] Rooijakkers S.H., van Kessel K.P., van Strijp J.A. (2005). Staphylococcal innate immune evasion. Trends Microbiol..

[48] Bardoel B.W., van der Ent S., Pel M.J., Tommassen J., Pieterse C.M., van Kessel K.P., van Strijp J.A. (2011). Pseudomonas evades immune recognition of flagellin in both mammals and plants. PLoS Pathog..

[49] Laarman A.J., Bardoel B.W., Ruyken M., Fernie J., Milder F.J., van Strijp J.A., Rooijakkers S.H. (2012). *Pseudomonas aeruginosa* alkaline protease blocks complement activation via the classical and lectin pathways. J. Immunol. (Baltim.).

[50] Bardoel B.W., Vos R., Bouman T., Aerts P.C., Bestebroer J., Huizinga E.G., Brondijk T.H., van Strijp J.A., de Haas C.J. (2012). Evasion of Toll-like receptor 2 activation by staphylococcal superantigen-like protein 3. J. Mol. Med. (Berl.).

[51] Gomery K., Muller-Loennies S., Brooks C.L., Brade L., Kosma P., Di Padova F., Brade H., Evans S.V. (2012). Antibody WN1 222–5 mimics Toll-like receptor 4 binding in the recognition of LPS. Proc. Natl. Acad. Sci. USA.

[52] Zhang S., Cheng K., Wang X., Yin H. (2012). Selection, synthesis, and anti-inflammatory evaluation of the arylidene malonate derivatives as tlr4 signaling inhibitors. Bioorg. Med. Chem..

[53] Zhang D., Li Y., Liu Y., Xiang X., Dong Z. (2013). Paclitaxel ameliorates lipopolysaccharide-induced kidney injury by binding myeloid differentiation protein-2 to block Toll-like receptor 4-mediated nuclear factor-kappab activation and cytokine production. J. Pharmacol. Exp. Ther..

[54] Piao W., Vogel S.N., Toshchakov V.Y. (2013). Inhibition of TLR4 signaling by tram-derived decoy peptides *in vitro* and *in vivo*. J. Immunol. (Baltim.).

[55] Takahashi M., Ota A., Karnan S., Hossain E., Konishi Y., Damdindorj L., Konishi H., Yokochi T., Nitta M., Hosokawa Y. (2012). Arsenic trioxide prevents nitric oxide production in lipopolysaccharide-stimulated raw 264.7 by inhibiting a TRIF-dependent pathway. Cancer Sci..

[56] Coburn B., Sekirov I., Finlay B.B. (2007). Type III secretion systems and disease. Clin. Microbiol. Rev..

